# Essential thrombocythemia associated with fibrosis involving bilateral renal sinuses: A case report

**DOI:** 10.1016/j.radcr.2023.07.016

**Published:** 2023-07-25

**Authors:** Adeleh Dadkhah, Seyed Morteza Bagheri, Nima Rakhshankhah

**Affiliations:** a Department of Diagnostic Radiology, Iran University of Medical Sciences, Hemat Highway next to Milad Tower, Tehran, 14535, Iran; b Department of Radiology, Hasheminejad Kidney Center (HKC), Iran University of Medical Sciences, Tehran, Iran

**Keywords:** Thrombocythemia, Essential, Retroperitoneal fibrosis, Acute kidney injury, Spiral CT, Ultrasonography

## Abstract

Essential thrombocythemia (ET) is associated with an increased risk of thrombosis and autoimmune renal involvement. We report an extremely rare case of an acute kidney injury (AKI) in the presence of bilateral renal pelvises fibrosis in a patient with a proven diagnosis of ET. A 48-year-old male patient with a past medical history of mild chronic kidney disease and ET was admitted to our hospital with AKI. The patient discontinued his hydroxyurea treatment for the past 2 months and laboratory data showed increasing serum creatinine levels and platelet counts with increased renal sizes, severe hydrocalyx, and bilateral renal sinuses’ fibrosis in imaging. The patient started again on hydroxyurea therapy and showed improvement in all laboratory scales. ET and increased levels of platelet-derived growth factors could cause renal sinuses fibrosis and glomerulopathy. In ET patients with renal sinuses’ fibrosis and glomerulopathy, initiating cytoreductive therapy could improve the outcome.

## Case presentation

A 48-year-old man with a past medical history of essential thrombocythemia (ET) which was diagnosed 4 years ago for migrainous headaches, increased platelet counts, positive *JAK2* mutation, negative *BCR-ABL1*, and mild chronic kidney disease (CKD) was admitted to our hospital with symptoms of nausea, vomiting, abdominal pain, and muscle cramps. He did not have a similar episode before this. A complete workup was done for the patient. On physical examination, general subcutaneous edema without signs of organomegaly, lymphadenopathy, and skin rashes were noted. His blood pressure was mildly elevated at 150/90. A review of his laboratory tests showed worsening serum creatinine levels in the past 6 months from 1.6 to 4.5 mg/dL (normal range = 0.6-1.1 mg/dL). Also increased platelet counts from 438 to 1954 × 10^3^/µL (normal range = 150-400 × 10^3^/ µL) was noted. The patient had no history of arterial or venous thrombotic events. The patient was on aspirin (75 mg once a day) and hydroxyurea (500 mg twice a day) therapy for the past 4 years from ET diagnosis, but recently from 2 months ago, he discontinued his hydroxyurea medication by himself. In other laboratory tests, mild anemia (11.5 mg/dL, normal range=13.8-17.2 g/dL), increased potassium levels (5.7 mEq/L, normal range = 3.5-5.2 mEq/L), and increased BUN levels (40 mg/dL, normal range = 20 mg/dL) were noted. Urinary analysis showed no hematuria or proteinuria.

**Fig. 1(A, C):**
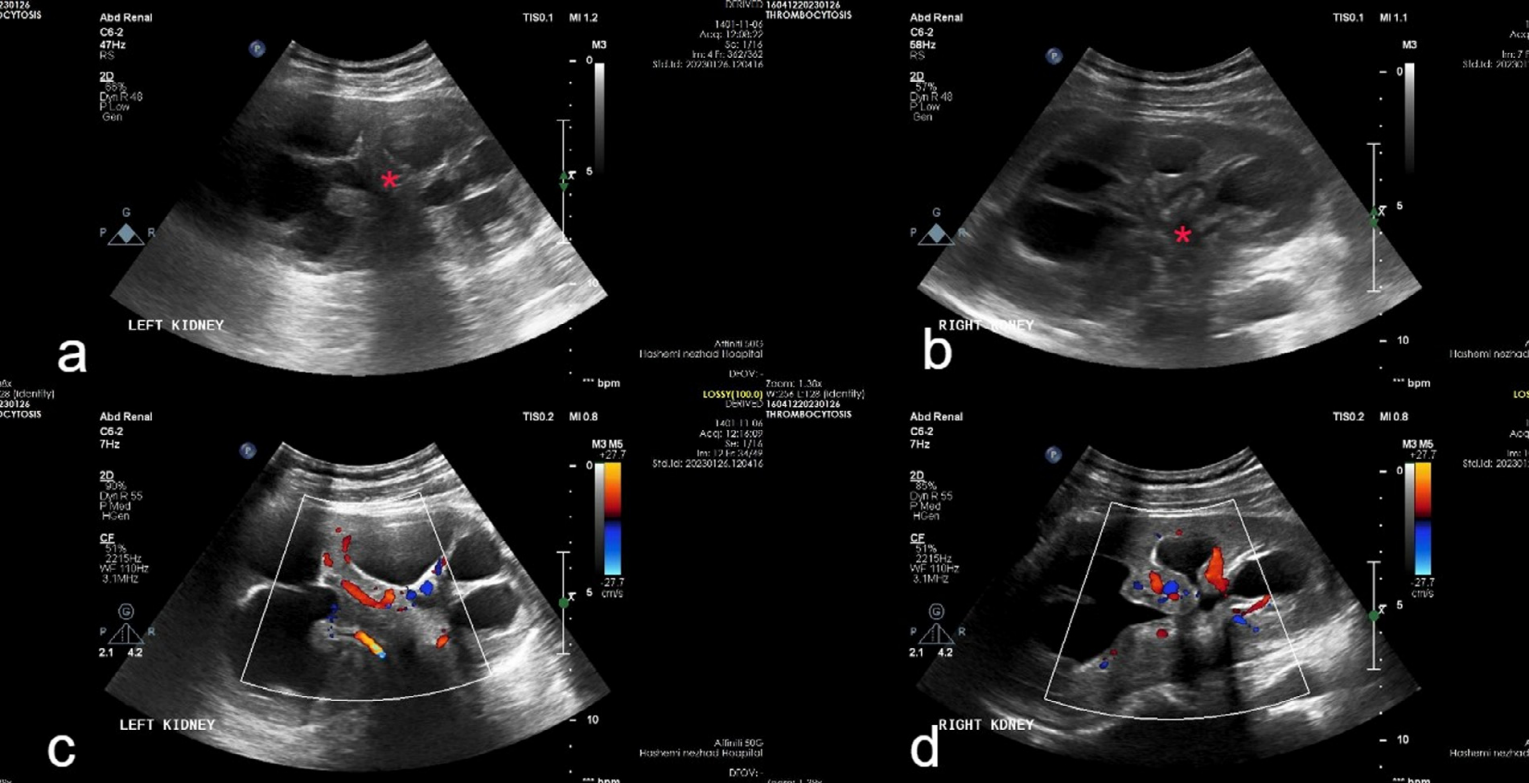
Urinary ultrasonographic examination of the left kidney shows dilated calyxes with echogenic fibrotic tissue in the renal pelvis. (B, D) Also, the same findings are seen in the right kidney.

**Fig. 2(A, B):**
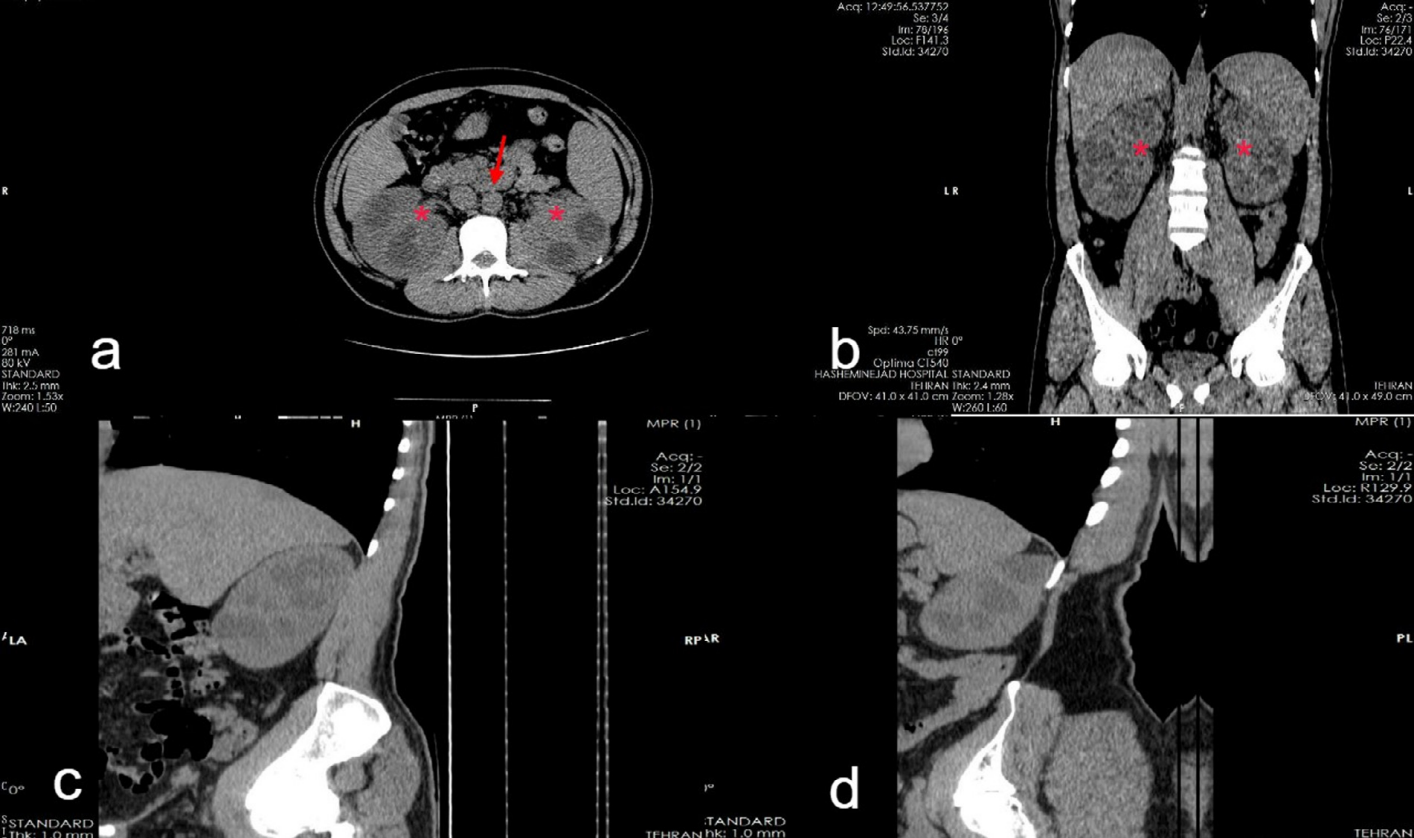
The nonenhanced CT scan shows diffuse severe hydro calyx in both kidneys with collapsed renal pelvises due to renal sinuses fibrotic tissue (*), also normal appearance retroperitoneum (arrow) is noted. (C) Curved sagittal view of right kidney with severe diffuse hydro calyx. (D) Curved sagittal view of left kidney with severe diffuse hydro calyx.

Ultrasonography was done for the patient and surprisingly revealed increased renal sizes, increased parenchymal echogenicity, severe diffuse hydrocalyx, and collapsed bilateral renal pelvises. A closer look at ultrasonography images revealed the fibrotic appearance of renal sinuses with the normal appearance of other retroperitoneal structures (Fig. 1). For further evaluation, a nonenhanced CT scan was done for the patient, and confirmed all ultrasonographic findings including renal sinuses’ fibrosis, severe bilateral hydrocalyx, increased renal sizes, and normal appearance of other retroperitoneal structures (Fig. 2).

The patient did not agree to undergo a renal biopsy. Therefore, a pathological evaluation could not be done. Hydroxyurea (500 mg twice daily) is started again for the patient. In the following first month of treatment, the patient was monitored for serum creatinine levels and platelet counts. Fortunately, all monitored laboratory values showed improvement in their values. Follow-up ultrasonography after one year showed persistent renal sinuses’ fibrosis with stable laboratory values in comparison to the first month of hydroxyurea retreatment.

## Discussion

Essential thrombocythemia is classified as myeloproliferative neoplasm (MPN) which causes an increased number of platelets in blood circulation [1]. According to the latest WHO classification, ET diagnosis requires 4 major criteria of platelet count ≥ 450 × 10^3^/µL, megakaryocyte lineage proliferation without significant left-shift of neutrophil granulopoiesis or erythropoiesis in bone marrow biopsy, presence of *JAK2, CALR, MPL* mutation or clonal marker, or absence of reactive thrombocytosis, and not meeting WHO criteria for other myeloid neoplasms [2,3]. ET patients are known for their higher risk of thrombotic events in comparison to the normal population but there are also lesser-known associations between ET and other conditions [4]. Various forms of autoimmune renal involvements have been reported in patients with ET including IgA nephropathy, fibrillary glomerulonephritis, and focal segmental glomerulosclerosis (FSGS) [5], [6], [7].

The prevalence of glomerulonephropathy and renal sinuses’ fibrosis in patients with MPNs is not well understood and is rarely seen. All the previous studies had described a few forms of glomerulopathy related to MPNs but none of them point out renal sinuses’ fibrosis. Cases of polycythemia vera (PV) showing proteinuria with FSGS appearance at renal biopsy were noted in previous studies [7,8]. Also, some cases of MPN-related nephropathies showed IgA nephropathy in pathologic assessments [5,9]. A probable pathogenic relation between ET/MPN and glomerular disease could be related to platelet-derived growth factor (PDGF) and its role in fibrotic processes. Peixoto et al. and Kanazawa et al. pointed out the correlation between PDFG and fibrotic processes in their studies [10,11]. Also, Stein-Oakley et al. described the higher expression of PDGF in glomeruli of patients with IgA nephropathy and FSGS disease [12].

In the present case, discontinuation of hydroxyurea treatment caused disease flare, which showed itself as increased platelet counts, increased serum creatinine levels, and renal sinuses fibrosis. As we noticed in our case, improvement in the laboratory findings with stable ultrasonographic findings was achieved after starting hydroxyurea. Interestingly, the use of hydroxyurea in ET has been found to decrease the levels of platelet counts and reduce inflammatory and oxidative stress markers which may cause a significant role in the treatment of thrombocythemia and related fibrosis [13,14].

In consideration of the significance of PDGF in the pathogenesis of fibrosis and glomerulopathy, cytoreductive therapies such as hydroxyurea, busulfan, anagrelide, ruxolitinib, and interferon could act an important role in treatment [15].

## Conclusions

In the case described above, a known ET patient had shown bilateral renal sinuses’ fibrosis and renal impairment, which showed significant improvement after hydroxyurea treatment. This could be an opening for further studies of ET, its relation to fibrosis, and above all the importance of cytoreductive treatment in the disease process.

## Author contributions

N.R. and A.D. wrote the main manuscript text and SM.B. prepared Figures 1 and 2. All authors reviewed the manuscript.

## Data availability

The datasets used and/or analyzed during the current study are available from the corresponding author upon reasonable request.

## Ethics approval and consent to participate

The patient gave written informed consent to participate.

## Patient consent

I declare that the patient is fully informed about the research and give me permission in full consciousness to use photographs, clinical and laboratory data of patient. Written informed consent is taken from patient.
